# High-dose chloroquine is metabolically cardiotoxic by inducing lysosomes and mitochondria dysfunction in a rat model of pressure overload hypertrophy

**DOI:** 10.14814/phy2.12413

**Published:** 2015-07-07

**Authors:** Antoine H Chaanine, Ronald E Gordon, Mathieu Nonnenmacher, Erik Kohlbrenner, Ludovic Benard, Roger J Hajjar

**Affiliations:** 1 Cardiovascular Institute, Mount Sinai School of MedicineNew York, New York; 2 Pathology Department, Mount Sinai School of MedicineNew York, New York

**Keywords:** Pathological hypertrophy, heart failure, autophagy, apoptosis, chloroquine and 3 methyladenine

## Abstract

Autophagy, macroautophagy and chaperone-mediated autophagy (CMA), are upregulated in pressure overload (PO) hypertrophy. In this study, we targeted this process at its induction using 3 methyladenine and at the lysosomal level using chloroquine and evaluated the effects of these modulations on cardiac function and myocyte ultrastructure. Sprague–Dawley rats weighing 200 g were subjected to ascending aortic banding. After 1 week of PO, animals were randomized to receive 3 methyladenine versus chloroquine, intraperitoneally, for 2 weeks at a dose of 40 and 50 mg/kg/day, respectively. Saline injection was used as control. Chloroquine treatment, in PO, resulted in regression in cardiac hypertrophy but with significant impairments in cardiac relaxation and contractility. Ultrastructurally, chloroquine accentuated mitochondrial fragmentation and cristae destruction with a plethora of autophagosomes containing collapsed mitochondria and lysosomal lamellar bodies. In contrast, 3 methyladenine improved cardiac function and attenuated mitochondrial fragmentation and autophagososme formation. Markers of macroautophagy and CMA were significantly decreased in the chloroquine group; whereas 3 methyladenine treatment significantly attenuated macroautophagy with a compensatory increase in CMA. Furthermore, chloroquine accentuated PO induced oxidative stress through the further decrease in the expression of manganese superoxide dismutase; whereas, 3 MA had a completely opposite effect. Taken together, these data suggest that high-dose chloroquine, in addition to its effect on the autophagy-lysosome pathway, significantly impairs mitochondrial antioxidant buffering capacity and accentuates oxidative stress and mitochondrial dysfunction in PO hypertrophy; highlighting, the cautious administration of this drug in high oxidative stress conditions, such as pathological hypertrophy or heart failure.

## Introduction

Autophagy is a cellular process involved in the recycling of damaged proteins and organelles in the cell (Klionsky and Codogno 2013; Jin and Klionsky 2014). A number of groups have shown that macroautophagy and its subtype, mitophagy, is upregulated in the heart in response to stressors such as pressure overload (PO) (Dorn 2009; Chaanine et al. 2012). We have shown that macroautophagy is upregulated in vitro*,* as early as two hours in phenylephrine-treated cardiac myocytes, as well as in vivo, 1 week after pressure overload hypertrophy (POH) (Chaanine et al. 2012). Moreover, a few days after induction of POH there is activation of the unfolded protein response (UPR) and chaperone-mediated autophagy due to the increases in the misfolded proteins and endoplasmic reticulum (ER) stress (Eskelinen et al. 2002; Ogata et al. 2006; Ding et al. 2007). We found that JNK is a dominant regulator of stress-induced mitophagy in POH via the post-translational modification and thus the activation of the transcription factor Foxo3a for the induction of its effector, BNIP3, which induces mitophagy in a noncanonical order as well as mitochondrial apoptosis (Chaanine et al. 2012, 2013). Therefore, JNK is an indirect marker of mitochondrial death and induces mitochondrial apoptosis and mitophagy through BNIP3. We showed for the first time that 3-methyladenine, in addition to its inhibitory effect on the canonical activation of autophagy via the inhibition of phosphatidylinositol 3-kinase (PI3K) class 3, attenuates JNK signaling leading to the decreased transcription of BNIP3 gene in POH and thus inhibits mitophagy as well as apoptosis (Chaanine et al. 2012, 2013). Since both types of autophagy, macroautophagy and chaperone-mediated autophagy, are activated in POH, the aim of this study was to understand the effect of modulating this process at two different sites. The first one being at the level of its induction using 3 methyladenine and the second one being at the level of the lysosome using chloroquine. Moreover, it has been reported that chloroquine induces mitochondrial dysfunction in cardiomyocytes (Essien and Ette 1986), however, the mechanism involved has not been clearly identified. We found that these drugs, beside their effect on the autophagy-lysosome pathway, have significant effect on the mitochondrial antioxidant buffering capacity. The clinical impact of this work lies in the fact that these compounds, especially chloroquine and hydroxychloroquine, are currently being evaluated as potential therapies for cancer (Cheong et al. 2012). There are a number of ongoing clinical trials studying chloroquine or hydroxychloroquine, in combination with other chemotherapeutic drugs, for the treatment of solid tumors (Cufi et al. 2013; Barnard et al. 2014; Mahalingam et al. 2014; Rangwala et al. 2014, 2014; Rosenfeld et al. 2014; Vogl et al. 2014). Moreover, chloroquine and its derivative hydroxychloroquine have long been used for the treatment of malaria and other rheumatologic diseases, and their associated skin manifestations, such as rheumatoid arthritis and systemic lupus erythematosus. Also, chloroquine toxicity induced cardiomyopathy has been reported in the literature (Roos et al. 2002; Soong et al. 2007). We used high doses of chloroquine in this study to modulate the autophagic activity similar to what is currently being used in cancer therapy (up to 1200 mg per day of chloroquine) and not in the treatment of malaria or other rheumatologic diseases where a much lower dose of chloroquine is usually used.

## Materials and Methods

### Experimental model of ascending aortic banding and study design

All procedures involving the handling of animals were approved by the Animal Care and Use Committee of the Mount Sinai School of Medicine and adhered with the Guide for the Care and Use of laboratory Animals published by the National Institutes of Health. The aortic banding model was used to generate pressure overload-induced hypertrophy and heart failure. Sprague–Dawley rats weighing 180–200 g underwent ascending aortic banding (AAB), as previously described in detail (Del Monte et al. 2002). PO developed right after the placement of the vascular clip. One week after AAB, echocardiography was performed to verify the hypertrophy phenotype and the presence of PO and animals were randomized to receive intraperitoneal injection of saline (*n* = 6), chloroquine (*n* = 6) or 3 methyladenine (*n* = 6). Age-matched sham operated animals were used as control (*n* = 4). The dose of chloroquine (Sigma, St. Louis, MO) and 3 methyladenine (Sigma) used were 50 mg/kg/day and 40 mg/kg/day, respectively. Each dose was dissolved in 0.5 mL of Saline. The HF study was aborted due to the toxicity seen with chloroquine administration in HF. Two weeks after treatment, the animals underwent echocardiography followed by a terminal hemodynamic evaluation procedure.

### Echocardiography

Transthoracic echocardiography was performed using a vivid seven echocardiography apparatus with a 14 MHZ probe (i13L probe, General Electric, New York, NY). Animals were sedated with ketamine 80–100 mg/kg injected intraperitoneally. Long axis parasternal views and short-axis parasternal two-dimensional (2D) views, at the mid-papillary level, of the left ventricle (LV) were obtained to calculate the LV end diastolic and end systolic volumes as well as the ejection fraction of the LV. Volumes were calculated by using the formulae of the area length method (V = 5/6 × A × L, where V: is the volume in ml, A: is the cross-sectional area of the LV cavity in cm^2^, obtained from the mid-papillary short parasternal image in diastole and in systole, and L: is the length of the LV cavity in cm, measured from the long parasternal axis image as the distance from the endocardial LV apex to the mitral-aortic junction in diastole and in systole). M-mode images were obtained by 2D guidance from the parasternal short axis view for the measurements of LV wall thickness of the septum and the posterior wall, LV end diastolic diameter, and LV end systolic diameter as well as to calculate the LV fractional shortening.

### Invasive pressure-volume loop measurements

At end point, LV pressure-volume loop measurements were obtained as previously described (Pacher et al. 2008). Hemodynamic recordings were performed after 5 min of stable heart rate. Anesthesia was maintained at 0.75–1% isoflurane to keep the animal sedated and maintain a stable heart rate around 350 beats/min. Hemodynamics were recorded after 5 min of stable heart rate using a Scisense P-V Control Unit (FY897B). The intrathoracic inferior vena cava was transiently occluded to decrease venous return during the recording to obtain load-independent P–V relationships. Linear fits were obtained for end-systolic pressure–volume relationships (ESPVR). At the end of the experiment, 50 *μ*L of 30% NaCl were slowly injected into the external jugular vein for ventricular parallel conductance (Gp) measurement as previously described (Pacher et al. 2008; Porterfield et al. 2009). Blood resistivity was measured using a special probe (Scisense). Volume measurements were initially obtained as blood conductance and calibrated, using the Baan equation (Baan et al. 1984) and pressure sensors were calibrated as per manufacturer’s instructions.

### Immunoblotting

Protein extraction was obtained by lysing 0.2 g of mechanically crushed tissue in a RIPA lysis buffer containing protease inhibitor (Roche Diagnostics Inc., Indianapolis, IN) and phosphatase inhibitor (Sigma-Aldrich, St. Louis, MO). Protein extracts for Western blotting were obtained by centrifuging the protein lysate at 10,000 *g* for 10 min at 4°C and then aspirating the supernatant. Protein concentrations were measured using the Bradford protein assay (Bio-Rad, Hercules, CA). About 30 *μ*g of proteins from each sample was loaded and electrophoresed using SDS-PAGE gels and then transferred to a PVDF membrane. The membrane was blocked for 1 h using blocking solution containing 0.15 mol/L sodium chloride, 3 mmol/L potassium chloride, 25 mmol/L tris-base, 5% skim milk, and 0.05% tween-20. Blots were incubated with the primary Ab overnight at 4°C. The following primary antibodies were used: GAPDH and LAMP-2 (Sigma; 1:10,000 and 1:1000 dilutions, respectively), HSC-70 (Abcam, Cambridge, MA, 1:1000 dilution), LC 3, BNIP3, p-eIF2a, eIF2a, BIP, JNK and Caspase 3/cleaved caspase 3 (Cell signaling, Danvers, MA, 1:1000 dilution), MnSOD (Enzo life sciences, Farmingdale, NY, (1:1000 dilution) and p-JNK (Promega, Madison, WI, 1:2000 dilution). The second day, after three washing steps with TBS-0.05% Tween-20, the blot was incubated with secondary horseradish peroxidase conjugated antibody (Thermo Scientific, Barrington, IL, 1:10,000 dilution) for 45 min. The blot was washed three times with TBS – 0.05% Tween-20 then a super signal west pico chemiluminescent substrate (Thermo Scientific, Barrington, IL) was used for the detection of protein bands, using the film method. Band densities were quantified using Photoshop program and were normalized to GAPDH to correct for variations in protein loading.

### Transmission electron microscopy

Fractions, about 1 mm^3^, from fresh ventricles were prefixed in a solution of 3% glutaraldehyde overnight at 4°C, postfixed in 1% osmium tetroxide (OsO4), dehydrated in an ascending series of alcohols, and embedded in epoxy resin. Ultrathin sections were stained with uranyl acetate and lead citrate. Samples were viewed under a transmission electron microscope (HITACHI H-7650, Tokyo, Japan). Images were taken at 1K, 5K, 12K and sometimes at 30K X magnification. Image J was used to measure the average mitochondrial area.

### Autophagic Flux experiment and reactive oxygen species (ROS) measurement

The autophagic flux experiment was performed in HEK 293 cells transfected with an eGFP-LC3 plasmid (Addgene, Cambridge, MA). Twenty-four hours after transfection, cells were starved in DMEM without serum for 12 h. 3 methyladenine (Sigma) and chloroquine (Sigma) were added at the time of starvation to inhibit autophagy induction and autophagosome–lysosome fusion at a concentration of 2 mmol/L and100 *μ*mol/L, respectively. HEK 293 cells cultured in DMEM with 10% FBS were used as control (CTL). Images were acquired 12 h after starvation using fluorescent microscope, Olympus 1 × 71. For the ROS measurement, 24 h after starvation, cells were treated with 10 *μ*mol/L dihydroethidium (DHE) (Sigma) for 30 min as previously described (Wojtala et al. 2014), and images were obtained using fluorescent microscope, Olympus 1 × 71. Cells cultured in DMEM with 10% FBS were used as CTL. This experiment was performed in two different cell lines, HEK 293, and AC-16 cells. DHE staining, in vivo*,* was performed by obtaining thin sections from frozen cardiac tissue, which were treated with 1 mmol/L dihydroethidium in PBS for 30 min at room temperature. Thereafter the sections were washed in PBS for 3 min × 3 and then mounted with DAPI. Images were taken immediately using fluorescent microscope, Olympus 1 × 71. Image J was used for the quantification of the fluorescent intensity.

### Statistical analysis

Results are shown as mean ± SD. Statistical significance was determined using Student–Newman–Keuls test. A *P*-value of <0.05 was considered statistically significant.

## Results

### Chloroquine group showed regression in cardiac hypertrophy, impaired myocardial relaxation, and depressed contractility

**Figure 1:**
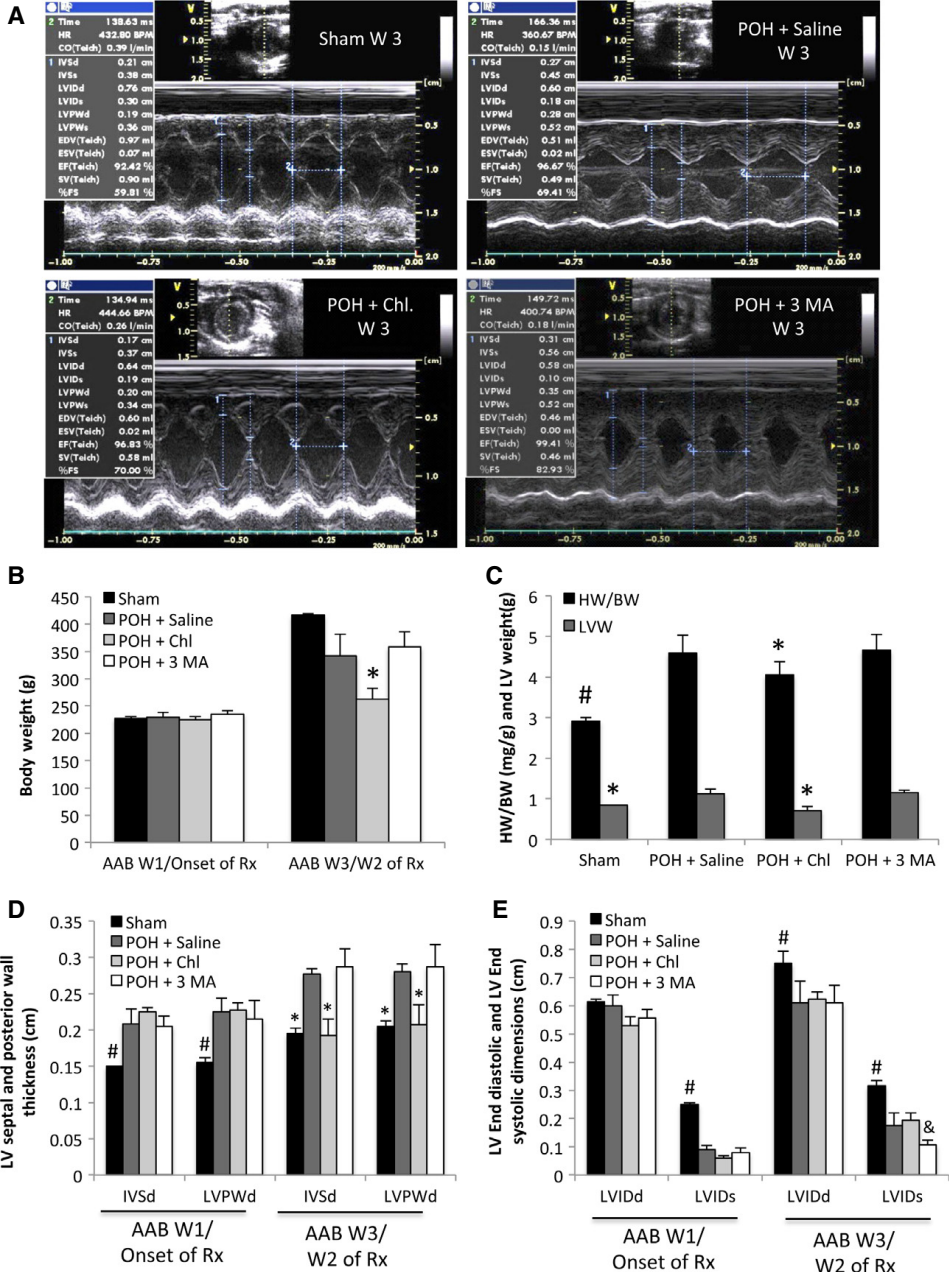
Chloroquine-treated animals had regression in cardiac hypertrophy compared to Saline and 3 Methyladenine (3 MA)-treated animals subjected to pressure overload. (A) Representative M-mode images of animals in each group. (B-C) Two weeks after treatment, the chloroquine-treated animals had significantly lower body weight, LV weight and heart weight to body weight ratio, *P < 0.05 versus POH + Saline and POH + 3 MA. The heart weight to body weight ratio significantly increased with pressure overload (PO), #*P* < 0.05 versus all other groups. (D) The LV septal and posterior wall thickness was significantly increased 1 week after PO, #*P* < 0.05 versus all other groups. However, 2 weeks after treatment, there was significant regression in these parameters in the chloroquine group, *P < 0.05 versus POH + Saline and POH + 3 MA. (E) The LV end systolic diameter was significantly lower in the POH groups 1 week after PO and the LV end diastolic diameter was significantly lower in the POH groups 3 weeks after PO, #*P* < 0.05 versus all other groups. Two weeks after treatment, the LV end systolic diameter was the lowest in the POH + 3 MA group, &*P* < 0.05 versus POH + Saline and POH + Chl. This reflected a significantly higher LV ejection fraction in the 3 MA group.

**Figure 2:**
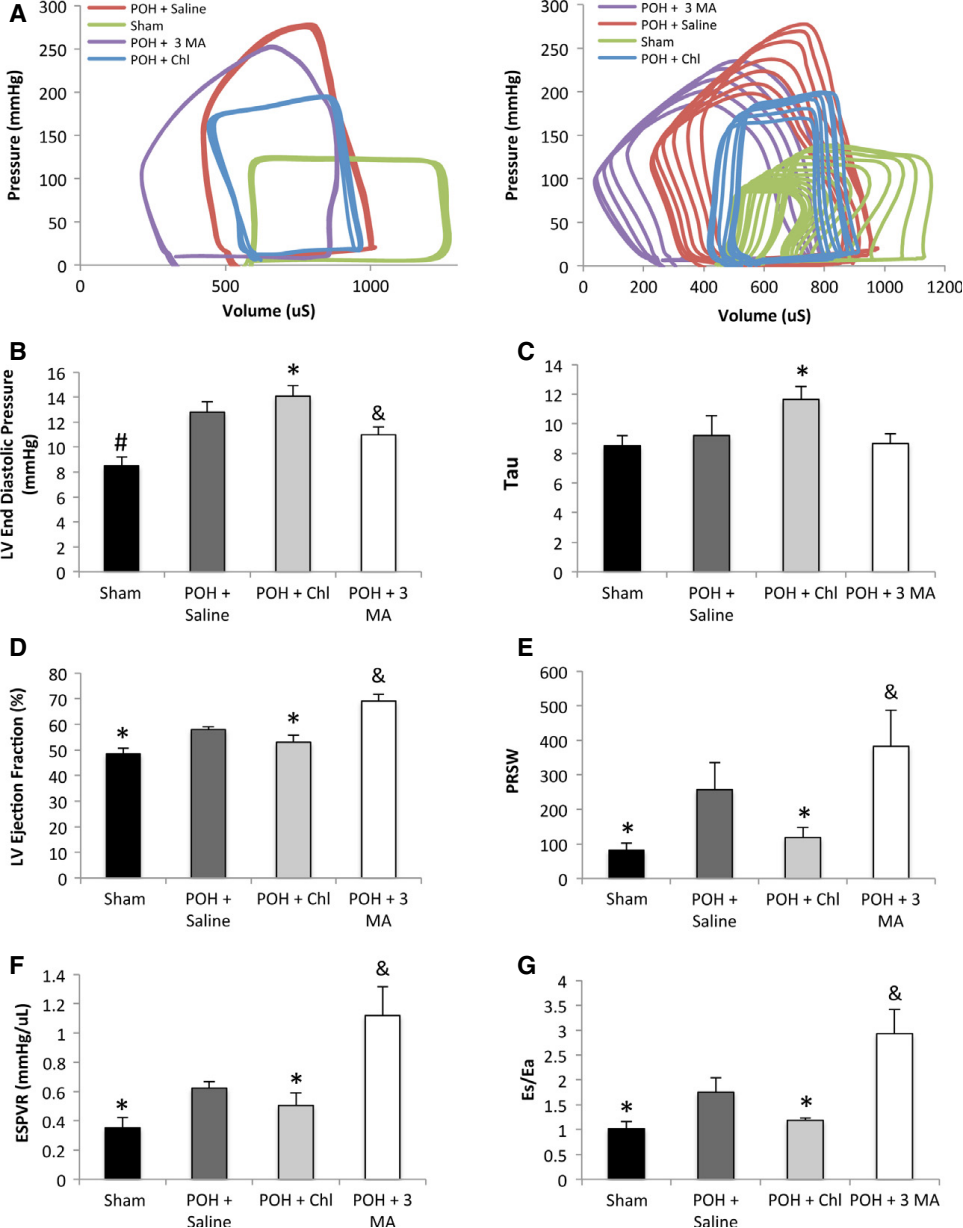
Chloroquine-treated animals had impaired myocardial relaxation, increased LV filling pressures and depressed contractility and efficiency compared to Saline and 3 Methyladenine-treated animals subjected to pressure overload. (A) representative, baseline and after inferior vena cava occlusion, pressure-volume loop tracings of animals in each group. (B–C) The LV filling pressure (LVEDP) was significantly increased 3 weeks after PO, #*P* < 0.05 versus all other groups. The chloroquine-treated animals had significantly increased LV end diastolic pressure and Tau despite that this group had the lowest LV maximum pressure, *P < 0.05 versus POH + Saline and POH + 3 MA. 3 Methyladenine-treated group had significantly lower LV end diastolic pressure, &*P* < 0.05 versus POH + Saline and POH + Chl. (D–G) LV ejection fraction and myocardial contractility measured by the preload recruitable stroke work (PRSW), end systolic pressure–volume relationship (Es or ESPVR) and myocardial efficiency (ESPVR/Ea) were significantly lower in the chloroquine-treated group, *P < 0.05 versus POH + Saline and POH + 3 MA, whereas these parameters were the highest in the 3 MA-treated group, &*P* < 0.05 versus POH + Saline and POH + Chl.

Echocardiographic data are shown in Figure1 and Table1. Representative M-mode images are shown in Figure1A. Two weeks after treatment, the chloroquine group had significantly lower body weight, LV weight, and heart to body weight ratio compared to the other POH groups Figure1B–C. There was regression in LV hypertrophy with significant decreases in the LV septal and posterior wall thicknesses in the chloroquine group compared to the other POH groups Figure1D. In contrast, the 3 methyladenine group had significantly lower LV end systolic diameters and volumes compared to the other two POH groups which reflected a significantly higher fractional shortening and ejection fraction Figure1E and Table1. Hemodynamic data are shown in Figure2 and Table2. Pressure–volume loop tracings, at baseline and during inferior vena cava occlusion, are shown in Figure2A. The chloroquine group had significantly higher LV end diastolic pressure despite that they had significantly lower LV maximum pressure, Figure2B. Moreover, Tau, which represents a time constant of LV pressure decay during the isovolumic relaxation period of the cardiac cycle, was significantly increased in the chloroquine group, Figure2C. The ejection fraction was slightly, but significantly lower in the chloroquine group, Figure2D. Parameters of contractility such as the preload recruitable stroke work (PRSW), the end systolic pressure–volume relationship (ESPVR or Es), and myocardial efficiency (ESPVR/Ea) were significantly reduced in the chloroquine group compared to the other groups, Figure2E–G. On the other hand, the 3 methyladenine-treated group had the lowest LV end diastolic pressures and the highest myocardial contractility and efficiency despite that this group had significantly higher LV maximum pressures than the chloroquine-treated group Figure2B–G and Table2.

**Table 1:** Echocardiography data of the pressure overload hypertrophy (POH) animals

	W1 post AAB/Onset of Rx				W3 post AAB/W2 of Rx			
Echo data	Sham (*n* = 4)	POH + Saline (*n* = 6)	POH + Chl (*n* = 6)	POH + 3 MA (*n* = 6)	Sham (*n* = 4)	POH + Saline (*n* = 6)	POH + Chl (*n* = 6)	POH + 3 MA (*n* = 6)
HW/BW (mg/g)					2.92 ± 0.08^A^	4.58 ± 0.45	4.05 ± 0.33^B^	4.66 ± 0.39
LVW (g)					0.83 ± 0.01^B^	1.12 ± 0.12	0.71 ± 0.11^B^	1.16 ± 0.05
BW (g)	227.5 ± 3.53	229.17 ± 9.33	225 ± 6.06	235.5 ± 6.44	385 ± 7.07	341.67 ± 40.29	263 ± 19.35^A^	358.17 ± 27.82
IVSd (cm)	0.15 ± 0.01^A^	0.21 ± 0.02	0.23 ± 0.01	0.21 ± 0.01	0.20 ± 0.01^B^	0.28 ± 0.01	0.19 ± 0.02^B^	0.29 ± 0.03
LVPWd (cm)	0.16 ± 0.01^A^	0.23 ± 0.02	0.23 ± 0.01	0.22 ± 0.03	0.21 ± 0.01^B^	0.28 ± 0.01	0.21 ± 0.03^B^	0.29 ± 0.03
LVIDd (cm)	0.62 ± 0.01	0.6 ± 0.03	0.53 ± 0.03	0.56 ± 0.03	0.75 ± 0.04^A^	0.61 ± 0.08	0.62 ± 0.03	0.61 ± 0.06
LVIDs (cm)	0.25 ± 0.01^A^	0.09 ± 0.01	0.06 ± 0.01	0.08 ± 0.02	0.32 ± 0.02^A^	0.18 ± 0.05	0.20 ± 0.03	0.11 ± 0.02^C^
LVFS (%)	59.22 ± 0.01^A^	85.14 ± 2.41	88.30 ± 1.52	85.63 ± 2.39	57.73 ± 0.57^A^	72.05 ± 6.64	68.83 ± 2.76	82.93 ± 2.20^C^
LVEDV (*μ*L)					568.13 ± 2.65^A^	434.58 ± 63.92	439.13 ± 69.14	415.45 ± 52.02
LVESV (*μ*L)					136.33 ± 1.88^A^	77.08 ± 19.69	84.08 ± 16.50	40.21 ± 10.77^C^
LVEF (%)					76 ± 0.44^A^	82.38 ± 3.39	80.82 ± 2.31	90.39 ± 1.88^C^

A *P* < 0.01 versus other groups.

B *P* < 0.01 versus POH + Saline and POH + 3 MA.

C *P* < 0.03 versus POH + Saline and POH + Chl

**Table 2:** Hemodynamic data of the pressure overload hypertrophy (POH) animals

Group	LV max. pressure (mmHg)	End diastolic pressure (mmHg)	End systolic pressure (mmHg)	Tau (Weiss)	LVEDV (*μ*L)	LVESV (*μ*L)	Ejection Fraction (%)	Heart Rate (bpm)	ESPVR (mmHg/*μ*L)	PRSW	ESPVR/Ea
Sham (*n* = 4)	127.33 ± 2.83^A^	8.5 ± 0.71^A^	124.83 ± 3.54	8.5 ± 0.71	467.02 ± 46.64	240 ± 14.14	48.50 ± 2.11^B^	388 ± 11.31	0.35 ± 0.07^B^	81.86 ± 21.04^B^	1.02 ± 0.14^B^
POH + Saline (*n* = 6)	204.6 ± 47.99	12.8 ± 0.84	124.07 ± 51.95	9.21 ± 1.34	519.68 ± 34.87	218.61 ± 17.74	57.96 ± 1.02	374.6 ± 16.09	0.62 ± 0.05	257.15 ± 77.54	1.75 ± 0.28
POH + Chl (*n* = 6)	189.5 ± 9.98	14.08 ± 0.83^B^	144.83 ± 18.19	11.64 ± 0.90^A^	514.27 ± 66.79	242.5 ± 43.49	53.04 ± 2.66^B^	376 ± 18.40	0.51 ± 0.09^B^	118.52 ± 29.9^B^	1.19 ± 0.05^B^
POH + 3 MA (*n* = 6)	267.87 ± 58.30^D^	10.98 ± 0.61^C^	167.82 ± 59.73	8.66 ± 0.68	483.24 ± 63.07	149.83 ± 26.86^A^	69.09 ± 2.62^A^	375.4 ± 20.99	1.12 ± 0.20^C^	382.37 ± 104.65^C^	2.93 ± 0.49^C^

A *P* < 0.02 versus other groups.

B *P* < 0.03 versus POH + Saline and POH + 3 MA.

C *P* < 0.02 versus POH + Saline and POH + Chl.

D *P* < 0.02 versus POH + Chl.

### Chloroquine significantly attenuated chaperone-mediated autophagy and macroautophagy in POH

**Figure 3:**
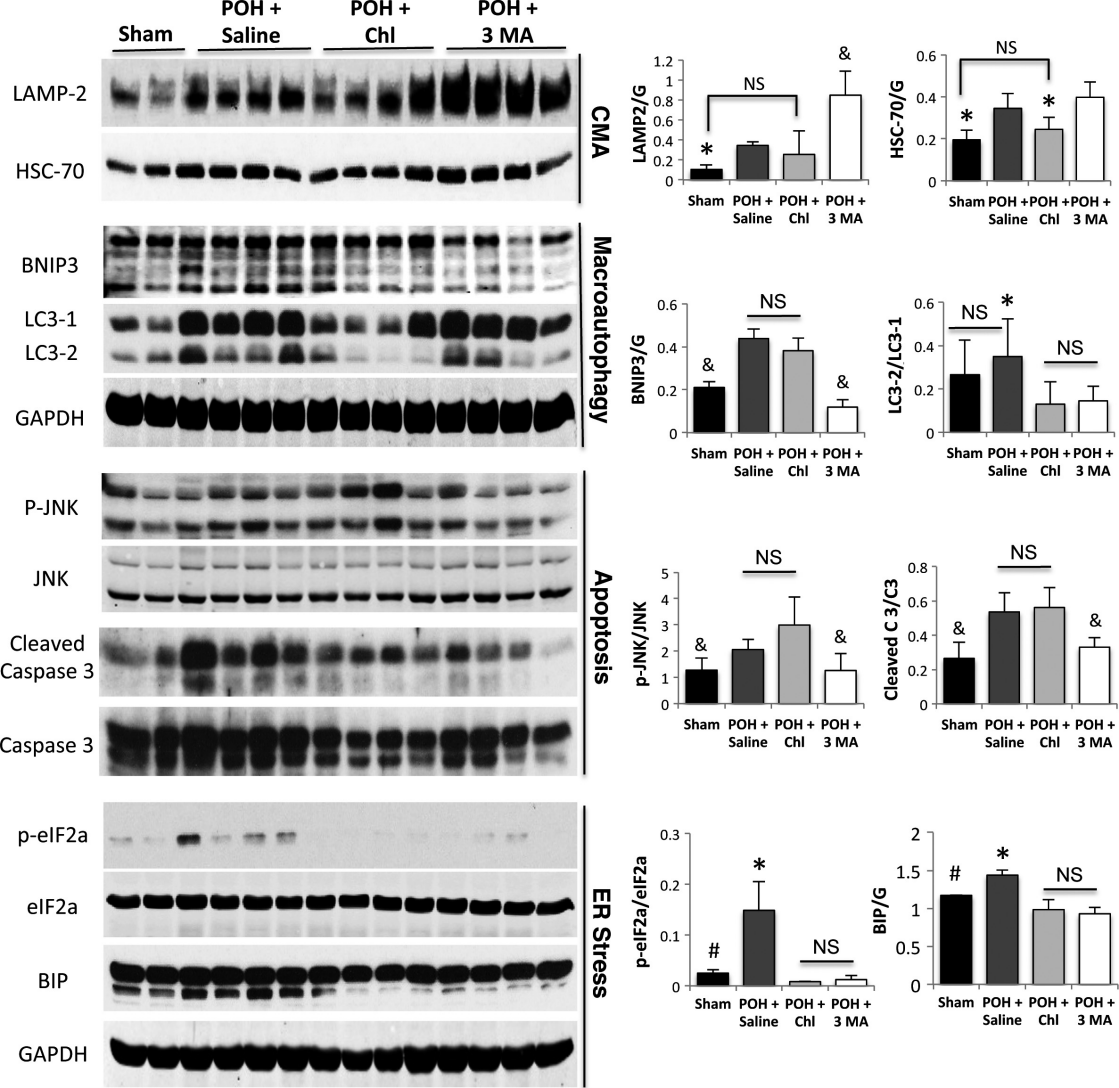
Markers of chaperone-mediated autophagy and macroautophagy were significantly attenuated in the chloroquine group; whereas, 3 MA attenuated macroautophagy and mitophagy with significant upregulation in chaperone-mediated autophagy. The lysosomal marker, LAMP-2 was increased in POH, but is robustly increased in the POH + 3 MA. There was significant decrease in LAMP-2 expression in the POH + Chl (except for the fourth sample), * and & *P* < 0.05 versus the other groups. The chaperone-mediated autophagy marker, HSC-70, was significantly increased in POH and was significantly decreased in the chloroquine group, *P < 0.05 versus POH + Saline and POH + 3 MA. The apoptotic markers, p-JNK and cleaved caspase 3, and the mitophagy and mitochondrial death marker, BNIP3, were significantly decreased in the 3 methyladenine group, &*P* < 0.05 versus POH + saline and POH + chloroquine. Although there were no significant differences in the ratio of LC3-2/LC3-1 between the sham and the POH + Saline groups, the expression of LC3-1 and its conversion to LC3-2 were increased in the POH + Saline group compared to sham. The ratio of LC3-2/LC3-1 was significantly decreased in the POH + Chl and POH + 3 MA groups, *P < 0.05 versus other POH groups, however in the former the expression of both LC3-1 and its conversion to LC3-2 were significantly decreased; whereas in the later the expression of LC3-1 was increased, however its conversion to LC3-2 was significantly decreased. The ER stress markers, BIP and p-eIF2a, were significantly increased in POH, #*P* < 0.05 versus POH + saline and were significantly decreased in the chloroquine and the 3 MA groups, *P < 0.05 versus other POH groups.

Western blotting of the LV tissue samples is shown in Figure3. The lysosomal marker (LAMP-2) and the chaperone-mediated autophagy marker (HSC-70) were significantly increased in POH. HSC-70 expression was significantly decreased in the chloroquine group. Similarly, LAMP-2 expression was significantly decreased in the chloroquine group, except for the fourth sample; whereas, these markers were significantly upregulated in the 3 Methyladenine group. The apoptotic markers (p-JNK and cleaved caspase 3) as well as the mitochondrial death and mitophagy marker (BNIP3) were significantly increased in the POH + saline and POH + chloroquine groups. 3 methyladenine treatment significantly attenuated the expression of these markers. The expression of LC3-1 and its conversion to LC3-2 was significantly increased in the POH + Saline group. Although the ratio of LC3-2/LC3-1 was significantly decreased in the chloroquine and the 3 methyladenine-treated groups, major difference does exist between both groups. In the former group, the expression of LC3-1 and its conversion to LC3-2 were significantly decreased; whereas, in the latter group the expression of LC3-1 was increased as in that of POH + Saline, however its conversion to LC3-2 was decreased. The unfolded protein response marker (BIP) and the ER stress marker (p-eIF2a) were significantly increased in POH. These parameters were significantly decreased in the chloroquine and the 3 methyladenine groups. Surprisingly, one would have expected that the ER stress markers to be increased in the chloroquine group, given that chaperone-mediated autophagy and macroautophagy were significantly downregulated in this group, further explanation is in the discussion section.

### Chloroquine accentuated mitochondrial fragmentation and cristae destruction in POH

**Figure 4:**
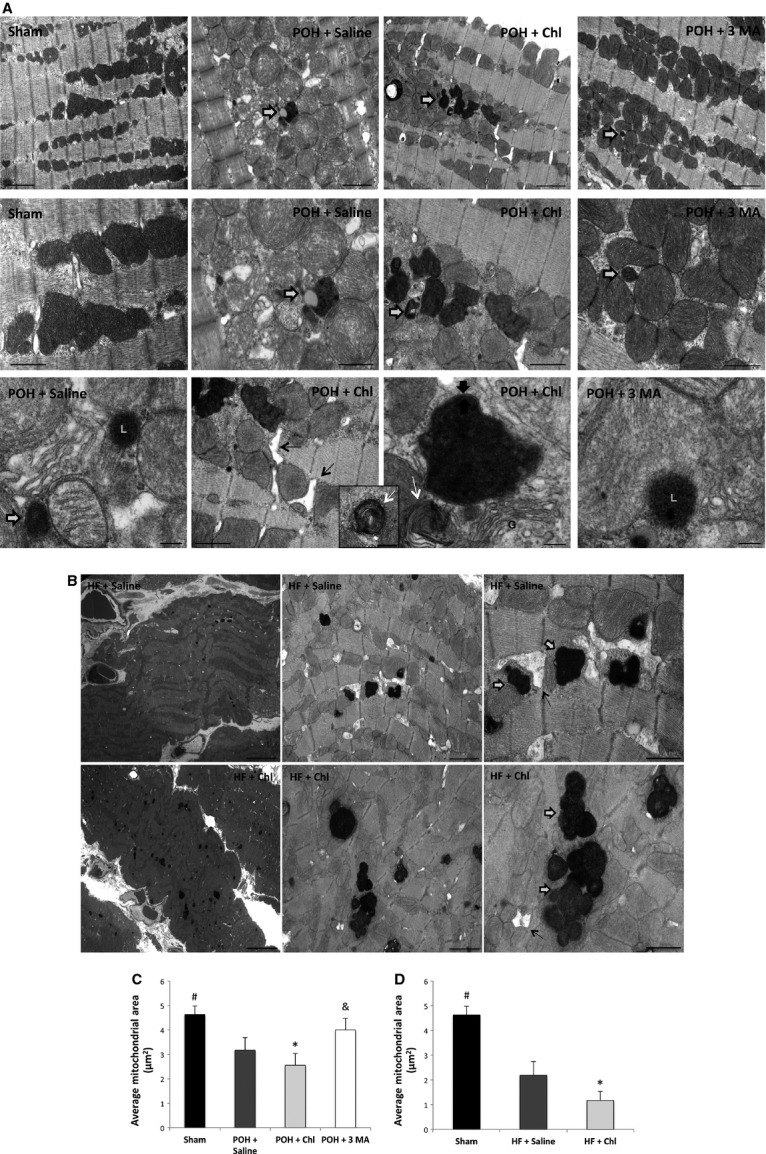
Ultrastructurally, chloroquine administration in POH accentuated mitochondrial fragmentation and cristae destruction. These effects of chloroquine were more pronounced in HF. (A and C) Mitochondrial fragmentation and cristae destruction were very well evident in the POH groups, #*P* < 0.05 versus other groups, with abundance in autophagosomes (white arrows with black contour) and lysosomes (L). The chloroquine group had the most significant ultrastructural findings. Mitochondrial fragmentation and cristae destruction, which were barely visible, were more pronounced, *P < 0.05 versus other POH groups. There were islands of collapsed mitochondria. Each one of those was surrounded by double membranes, which were seen on higher magnification (thick black arrows). The T-tubules were dilated (thin black arrows) and strikingly there was abundance of lysosomal lamellar or myelin bodies that represent aggregates of phospholipids and proteins (thin white arrows). On the contrary, the 3 Methyladenine group had the least mitochondrial damage and fragmentation, &*P* < 0.05 versus other POH groups. The cristae were dense and sharp looking. Also, there was abundance in lysosomes (L). G: golgi system or apparatus. The above row represents images 5K X magnified, scale bar 2 *μ*m. The middle row represents images 12K X magnified, scale bar 1 *μ*m and the bottom row represents images 30K X magnified (except for the second image from the left 12K X magnified), scale bar 0.25 *μ*m. (B) representative TEM images of HF animals treated with chloroquine versus Saline. (D) mitochondrial fragmentation was more pronounced when chloroquine was administered to animals with HF, much more than when chloroquine was administered in the context of POH, #*P* < 0.05 versus HF groups and *P < 0.05 versus HF + Saline. Images from left to right are 1K, 5K and 12K X magnified, Scale bar 10 *μ*m, 2 *μ*m and 1 *μ*m, respectively.

Representative transmission electron microscopy (TEM) images are shown in Figure4A. Ultrastructurally, mitochondrial fragmentation, Figure4C, and cristae destruction were very well evident in the POH + Saline-treated group compared to sham. Also, there was abundance of autophagosomes and lysosomes in this group. The chloroquine group had the most significant ultrastructural findings. There were accentuated mitochondrial fragmentation, Figure4C and cristae destruction to such a degree that they were barely visible. Islands of collapsed mitochondria could be seen in certain areas. Each collapsed mitochondrion was surrounded by double membranes, which were seen on higher magnification. The T-tubules were dilated and strikingly there was abundance of lysosomal lamellar or myelin bodies that represent aggregates of phospholipids and proteins. On the contrary, the 3 methyladenine group had the least mitochondrial fragmentation, Figure4C. The cristae were dense and sharp looking. Also, there was abundance of lysosomes in this group. The ultrastructural changes seen in the chloroquine group in POH were even more pronounced when chloroquine was administered in animals with HF, Figure4B and Figure4D. That part of the study had to be aborted as the animals became extremely sick. They lost over 70 grams within 2 weeks of chloroquine administration and were dehydrated and failed to thrive. Representative TEM images are shown in Figure4B.

**Figure 5:**
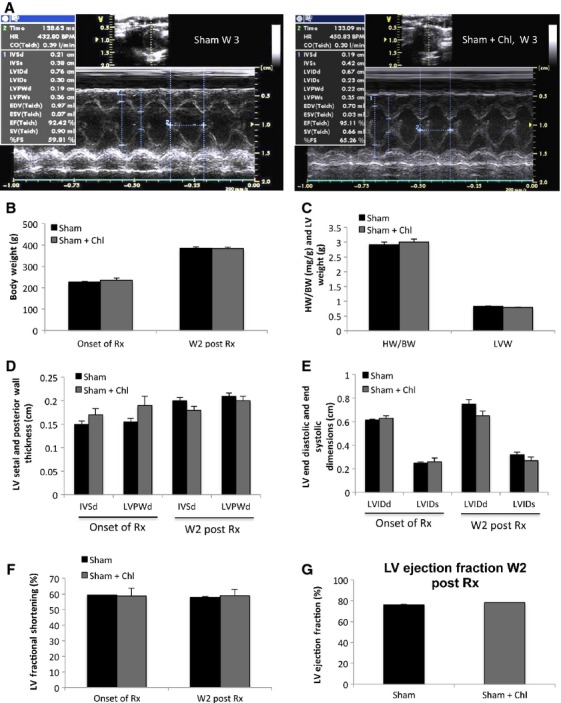
There were no differences in the echocardiographic parameters between the sham and sham plus chloroquine-treated animals. (A) representative M-mode images of animals in each group. (B–D) there were no differences in the body weight, LV weight and heart to body weight ratio as well as in the LV septal and posterior wall thickness between both groups. (E-G) No significant differences were noted in LV end diastolic and end systolic diameters, in fractional shortening and in ejection fraction between both groups.

**Figure 6:**
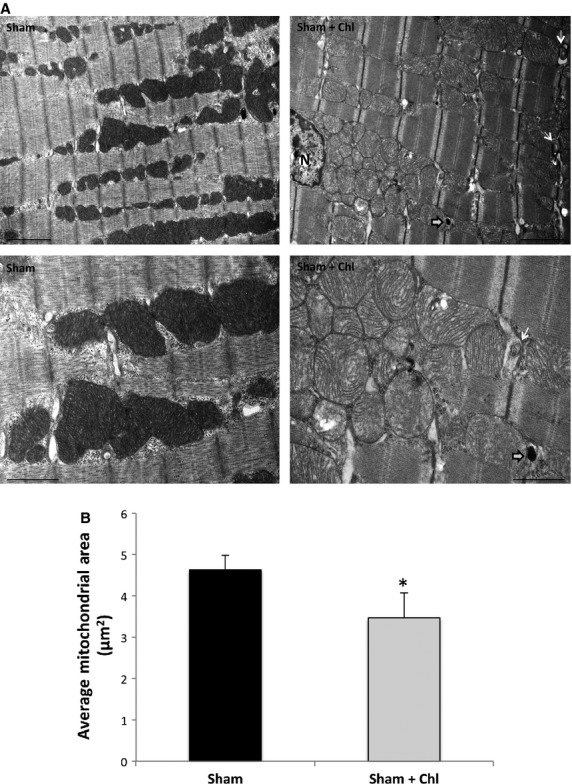
Mitochondrial fragmentation and cristae destruction were less pronounced, but significant, when chloroquine was administered to sham animals. (A, B) Fewer autophagosomes (white arrows with black contour) and myelin bodies (white arrows) were noted in the chloroquine-treated sham animals. Also, mitochondrial fragmentation and cristae destruction were less pronounced than the POH + Chl, but significant when compared to sham animals, *P < 0.05 versus sham group. Images 5K (upper row) and 12K (lower row) X magnified, scale bar 2 *μ*m and 1 *μ*m, respectively.

The administration of similar high-dose chloroquine in sham animals was well tolerated. There were no significant effects on body weight or heart weight to body weight and LV weight compared to sham animals. Functional data by echocardiography revealed no significant differences as well, Figure5 and Table3. Ultrastructurally, mitochondrial fragmentation and mitochondrial cristae destruction were less pronounced than the POH + Chl group but significant when compared to the sham group, Figure6A–B.

**Table 3:** Echocardiography data of the sham and sham + Chloroquine groups

	Onset of treatment		W2 of treatment	
Echo data	Sham (*n* = 4)	Sham + Chl (*n* = 4)	Sham (*n* = 4)	Sham + Chl (*n* = 4)
HW/BW (mg/g)			2.92 ± 0.08	3.01 ± 0.1
LVW (g)			0.83 ± 0.01	0.78 ± 0.02
BW (g)	227.5 ± 3.54	235 ± 11.18	385 ± 7.07	384.2 ± 5.63
IVSd (cm)	0.15 ± 0.01	0.17 ± 0.01	0.20 ± 0.01	0.19 ± 0.01
LVPWd (cm)	0.16 ± 0.01	0.19 ± 0.02	0.21 ± 0.01	0.2 ± 0.01
LVIDd (cm)	0.62 ± 0.01	0.63 ± 0.02	0.75 ± 0.04	0.65 ± 0.04
LVIDs (cm)	0.25 ± 0.01	0.26 ± 0.03	0.32 ± 0.02	0.27 ± 0.03
LVFS(%)	59.22 ± 0.01	58.63 ± 5.1	57.73 ± 0.57	58.97 ± 3.93
LVEDV (*μ*L)			568.13 ± 2.65	503.05 ± 45.49
LVESV (*μ*L)			136.33 ± 1.88	110 ± 10
LVEF (%)			76 ± 0.44	78.13 ± 0.12

No statistical significant difference between both groups 2 weeks after treatment.

### Chloroquine treatment impaired mitochondrial antioxidant buffering capacity and accentuated oxidative stress in POH

**Figure 7:**
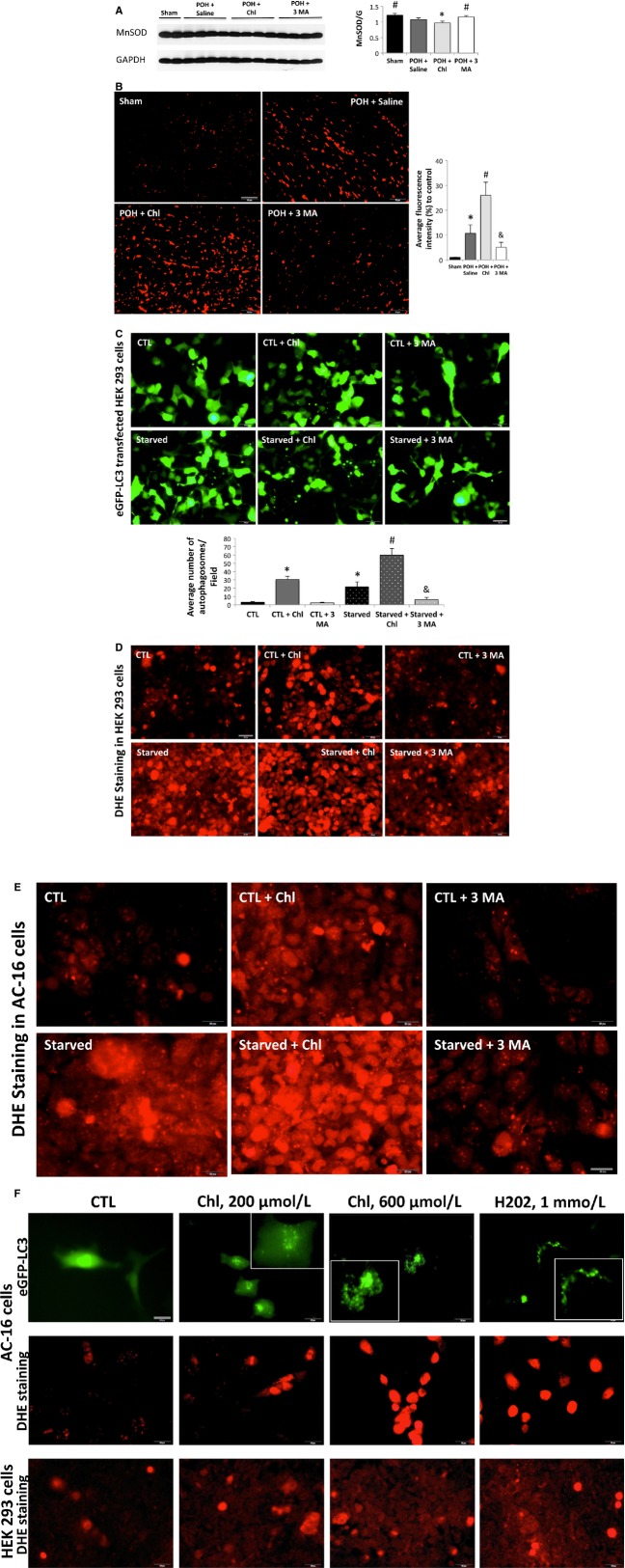
Chloroquine treatment impaired mitochondrial antioxidant buffering capacity and accentuated oxidative stress in POH; whereas, 3 MA had a completely opposite effect. (A) The expression of MnSOD was significantly decreased in POH, which was further decreased with chloroquine treatment, #*P* < 0.05 versus POH + Saline and POH + Chl and *P < 0.05 versus other groups. (B) DHE staining of cardiac tissue sections, *P < 0.05 versus sham, #*P* < 0.05 versus other groups and &*P* < 0.05 versus other POH groups. (C) Autophagic flux in HEK 293 cells starved for 12 h, *P < 0.05 versus CTL and CTL + 3 MA, #*P* < 0.05 versus all other groups and &*P* < 0.05 versus starved and starved + Chl. (D–E) ROS production was increased in starved and chloroquine-treated HEK 293 and AC-16 cells. Chloroquine further increased ROS in Starved cells; whereas, 3 MA significantly attenuated ROS during starvation. (F) There was a dose-dependent increase in autophagosomes and in ROS in HEK 293 and AC-16 cells treated with chloroquine for 3 h. H2O2 was used as a positive control. Results are representative of three independent experiments. Scale bar 50 *μ*m.

Given that the administration of high-dose chloroquine was associated with significant mitochondrial fragmentation and cristae destruction, and to further validate the transmission electron microscopy findings, we examined the effect of these drugs on oxidative stress, by assessing the amount of ROS using DHE staining, and their effect on the mitochondrial antioxidant buffering capacity, by assessing the expression of the mitochondrial-specific superoxide dismutase (MnSOD). Western blotting of LV samples showed significant decrease in MnSOD expression in POH, however, the administration of high-dose chloroquine in POH attenuated further the expression of MnSOD leading to further impairment in mitochondrial antioxidant capacity, Figure7A. On the other hand, there was significant increase in MnSOD expression and therefore improvement in mitochondrial antioxidant buffering capacity in the 3 MA group, Figure7A. DHE staining of heart tissue sections showed significant increases in ROS in PO which was further increased with chloroquine treatment. On the other hand, 3 MA significantly attenuated ROS and oxidative stress in POH, Figure7B. To further validate these results, we assessed autophagosome formation and ROS production in two different cell lines, HEK 293 and AC-16 cells, cultured in DMEM with 10% FBS (CTL) versus DMEM with no serum (starved), which was used as a stressor. Autophagososmes as well as ROS were significantly increased with starvation, which were further significantly increased in the starved + Chl-treated cells. Also, autophagosomes and ROS were significantly increased in the CTL + Chl compared to CTL and CTL + 3 MA-treated cells. On the other hand, 3 MA significantly attenuated the number of autophagosomes and ROS in starved cells, Figure7C–E. Moreover, there was a dose-dependent increase in ROS in chloroquine-treated cells, Figure7F.

## Discussion

Macroautophagy as well as chaperone-mediated autophagy are upregulated in the heart in oxidative stress conditions such as POH for the recycling of misfolded proteins and damaged organelles. We modulated the autophagy-lysosome pathway in vivo*,* using 3 methyladenine and chloroquine as mentioned above, and studied its effect on cardiac function in correlation with ultrastructural changes. Significant differences in cardiac function and ultrastructure were observed between the two groups. There was regression in cardiac hypertrophy in the chloroquine group but it was associated with worsening in cardiac function. Hemodynamic assessment of the chloroquine-treated animals revealed significantly higher LV filling pressures and impaired myocardial relaxation despite a lower LV maximum pressure in this group. Also, myocardial contractility was significantly reduced compared to the saline and 3 methyladenine-treated animals. Ultrastructurally, chloroquine treatment accentuated mitochondrial fragmentation and cristae destruction in POH. There were abundance of collapsed mitochondria, and sometimes even in the form of islands or series of damaged and collapsed mitochondria each surrounded by double membranes observed on higher magnification. Also, unique to the chloroquine group was the plethora of lysosomal lamellar bodies, which looked as concentric onion like arranged layers and represent aggregates of phospholipids and proteins. The lysosomal lamellar structures are well described, but are not specific to chloroquine therapy (Reasor 1989). The T-tubules were dilated and disorganized. These ultrastructural changes, except for the lysosomal lamellar bodies, also existed in the POH + Saline group but to a much less extent and severity. On the other hand, the 3 methyladenine-treated animals preserved their cardiac hypertrophy and systolic function as opposed to the chloroquine group. Hemodynamically, parameters of myocardial relaxation and contractility were significantly better in the 3 MA group compared to the other POH groups. Ultrastructurally, 3 methyladenine significantly attenuated mitochondrial fragmentation, cristae destruction and autophagosome formation. These effects of 3 methyladenine are partially related to the decreased expression of the mitochondrial death and mitophagy marker, BNIP3, as we showed earlier (Chaanine et al. 2012, 2013).

Molecular analysis of LV myocardial tissue by western blotting revealed that chloroquine treatment was associated with significant decreases in markers of both macroautophagy and chaperone-mediated autophagy, however the expression of the apoptotic and the death signaling markers were not significantly different compared to the POH + Saline group. Whereas 3 methyladenine treatment was associated with significant decreases in macroautophagy and apoptosis with a compensatory upregulation in chaperone-mediated autophagy as a rescue mechanism for the degradation of misfolded proteins, which has been shown as well by Kaushik et al. (2008). The UPR and ER stress markers were increased in POH + Saline group and significantly decreased in the chloroquine and 3 methyladenine groups. One would have expected that the inhibition of both forms of autophagy, which is the case of chloroquine therapy, would have been associated with increases in ER stress markers, which was not seen in our case. Possible explanations include the toxic effect of chloroquine at the DNA level leading to attenuation in gene expression or that these cells are mainly in the apoptotic phase, way beyond the ER stress phase, which is most likely the case here, however, this was not fully investigated in this study. On the other hand, the inhibition of macroautophagy by 3 methyladenine allows the cell to upregulate chaperone-mediated autophagy as a rescue mechanism for the degradation of misfolded proteins and thus leads to the attenuation in ER stress and in improved cell survival (Massey et al. 2006; Kaushik et al. 2008; Zhang and Cuervo 2008; Orenstein and Cuervo 2010).

**Figure 8:**
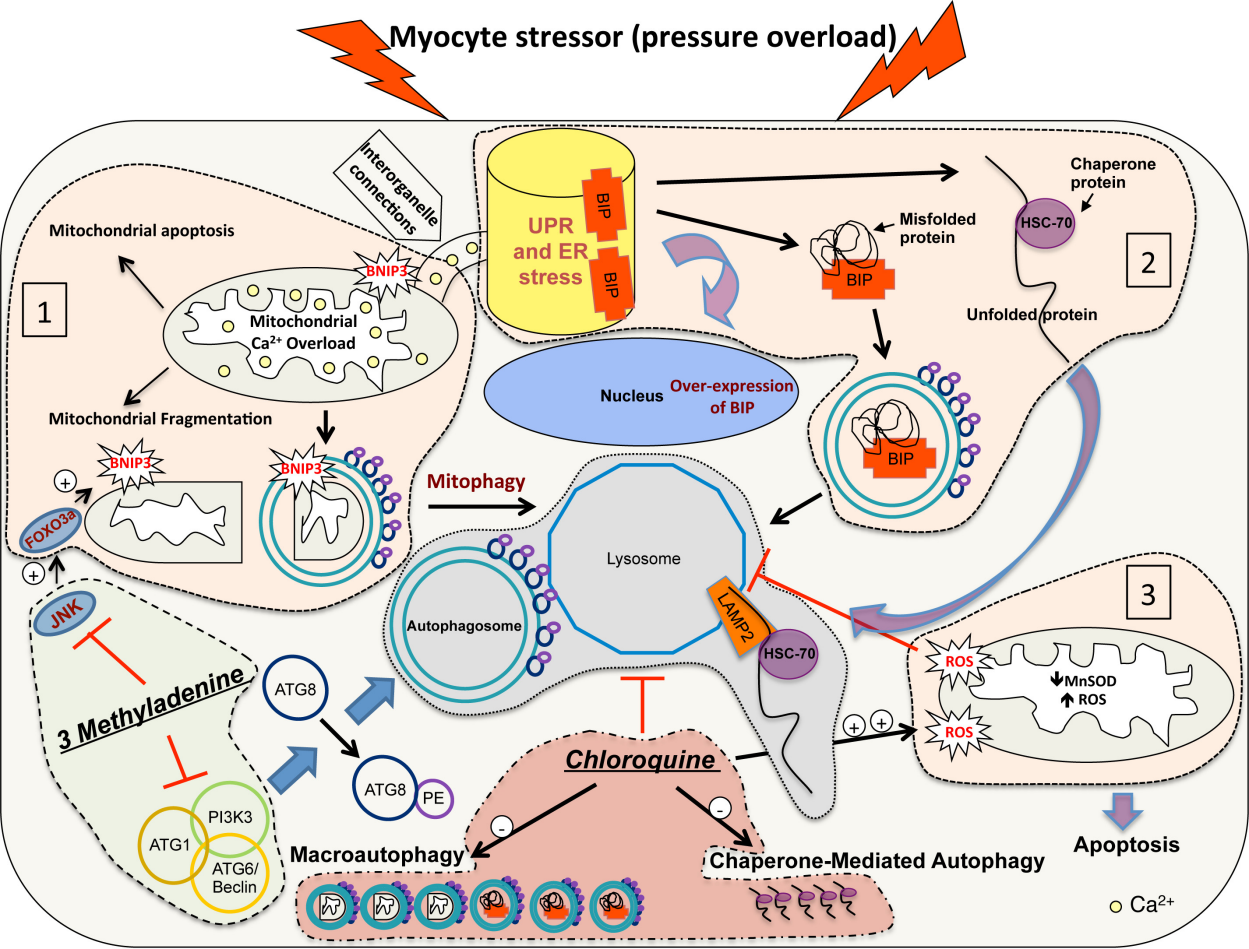
Schematic drawing highlighting the consequences of autophagy modulation in pressure overload. PO is an oxidative stress condition associated with: (1) increases in JNK activity and upregulation of the mitochondrial death and mitophagy marker, BNIP3, leading to mitochondrial calcium overload, mitophagy and mitochondrial apoptosis, (2) ER stress with increases in macroautophagy and CMA for the recycling of misfolded proteins and (3) impairment in mitochondrial antioxidant buffering capacity due to the decreases in the expression of MnSOD. These changes are highlighted in light pink. The lysosomes play a critical role and their integrity and function is quintessential for the recycling of the misfolded proteins and damaged organelles (highlighted in light gray). Chloroquine, a metabolically toxic drug, impairs lysosomal function and increases ROS production, which further perpetuates lysosomal and mitochondrial dysfunction and promotes apoptosis. On the other hand, 3 methyladenine inhibits macroautophagy with compensatory increases in CMA and lysosomal markers and attenuates JNK-BNIP3 signaling, ROS production and apoptosis (highlighted in light green). This highlights that the forces driving/modulating the autophagic process, rather than autophagy itself, are the ones that, at least to a certain extent, determine the fate of the cardiac myocyte. It also highlights that the administration of a metabolically toxic drug is deleterious in conditions of high oxidative stress. Of course, other processes take place in the context of PO and oxidative stress, which are not highlighted in this figure. ATG1: autophagy-related protein1, ATG6, or Beclin1, ATG8: autophagy-related protein 8, PI3K3: phosphatidylinositol 3-kinase class 3, PE: phosphatidylethanolamine.

In addition to their effect on the autophagy-lysosome pathway, we have found that these drugs affect the mitochondrial antioxidant buffering capacity and therefore mitochondrial function and oxidative stress. Chloroquine treatment accentuated oxidative stress and ROS in POH with significant decreases in the expression of MnSOD; whereas, 3 methyladenine had a totally opposite effect. This heightened state of oxidative stress observed in the chloroquine group results in the peroxidation of the lysosomal membrane leading to the instability of the lysosomes and to the accumulation of lipofuscin material and therefore further impairs lysosomal and cellular function and promotes apoptosis (Kurz et al. 2008; Terman et al. 2008). These results are highlighted in Figure8.

We believe that our data have clinical relevance, as there is an increase interest and ongoing clinical trials using chloroquine in combination with other chemotherapy drugs for the treatment of solid tumors. In the day-to-day clinical practice, it is increasingly common to come across patients who have malignancy with a co-existing cardiovascular disease. Also, there is more awareness that newer chemotherapy or investigational drugs are shown to have cardiovascular side effects or toxicity. For example we found that Bortezomib, which is an autophagy inducer and proteasome inhibitor used in the treatment of patients with multiple myeloma and prostate cancer, caused cardiovascular toxicity and even death when used in rats with PO induced pathological hypertrophy and heart failure that was not seen with similar doses in rats with normal hearts (Chaanine et al. 2014). Bortezomib in combination with chloroquine is being studied for the treatment of patients with refractory myeloma (Vogl et al. 2014).

In summary, the forces driving/modulating the autophagy-lysosome pathway, rather than autophagy itself, are the ones that determine the fate of the cardiac myocyte. For instance, high-dose chloroquine in POH is metabolically toxic by inducing lysosome dysfunction and by attenuating the mitochondrial antioxidant buffering capacity leading to a state of heightened oxidative stress, which further perpetuates lysosome and mitochondrial function and promotes apoptosis. Whether other deleterious effects of chloroquine exist, has not been evaluated in this study. On the other hand, 3 methyladenine improves cardiac function, despite its inhibitory effect on macroautophagy, through the downregulation of the mitochondrial death and mitophagy marker, BNIP3, and through the upregulation of CMA and the improvement in mitochondrial antioxidant buffering capacity, Figure8.

### Limitations

We understand that there is a limitation to the study in the sense that the drugs used might have off-target effects within or outside the heart; however, it is not feasible to identify all possible mechanisms involved in one study. In general, the data suggest that high-dose chloroquine is associated with cardiac toxicity and is poorly tolerated in situations of high oxidative stress, such as pressure overload hypertrophy, highlighting the cautious administration of this drug under these circumstances.

## Conflict of Interest

Roger Hajjar is a scientific founder of Celladon Co., which plans to commercialize AAV1.SERCA2a for the treatment of heart failure. Other authors have no conflict of interest.
